# Genome-wide homozygosity and risk of four non-Hodgkin lymphoma subtypes

**DOI:** 10.20517/jtgg.2021.08

**Published:** 2021-06-17

**Authors:** Amy Moore, Mitchell J. Machiela, Moara Machado, Sophia S. Wang, Eleanor Kane, Susan L. Slager, Weiyin Zhou, Mary Carrington, Qing Lan, Roger L. Milne, Brenda M. Birmann, Hans-Olov Adami, Demetrius Albanes, Alan A. Arslan, Nikolaus Becker, Yolanda Benavente, Simonetta Bisanzi, Paolo Boffetta, Paige M. Bracci, Paul Brennan, Angela R. Brooks-Wilson, Federico Canzian, Neil Caporaso, Jacqueline Clavel, Pierluigi Cocco, Lucia Conde, David G. Cox, Wendy Cozen, Karen Curtin, Immaculata De Vivo, Silvia de Sanjose, Lenka Foretova, Susan M. Gapstur, Hervè Ghesquières, Graham G. Giles, Martha Glenn, Bengt Glimelius, Chi Gao, Thomas M. Habermann, Henrik Hjalgrim, Rebecca D. Jackson, Mark Liebow, Brian K. Link, Marc Maynadie, James McKay, Mads Melbye, Lucia Miligi, Thierry J. Molina, Alain Monnereau, Alexandra Nieters, Kari E. North, Kenneth Offit, Alpa V. Patel, Sara Piro, Vignesh Ravichandran, Elio Riboli, Gilles Salles, Richard K. Severson, Christine F. Skibola, Karin E. Smedby, Melissa C. Southey, John J. Spinelli, Anthony Staines, Carolyn Stewart, Lauren R. Teras, Lesley F. Tinker, Ruth C. Travis, Claire M. Vajdic, Roel C. H. Vermeulen, Joseph Vijai, Elisabete Weiderpass, Stephanie Weinstein, Nicole Wong Doo, Yawei Zhang, Tongzhang Zheng, Stephen J. Chanock, Nathaniel Rothman, James R. Cerhan, Michael Dean, Nicola J. Camp, Meredith Yeager, Sonja I. Berndt

**Affiliations:** 1 Division of Cancer Epidemiology and Genetics, National Cancer Institute, Bethesda, MD 20892, USA.; 2 Departamento de Biologia Geral, Instituto de Ciências Biológicas, Universidade Federal de Minas Gerais, Belo Horizonte 31270-901, Brazil.; 3 Division of Health Analytics, City of Hope Beckman Research Institute, Duarte, CA 91010, USA.; 4 Department of Health Sciences, University of York, York YO10 5DD, UK.; 5 Department of Health Sciences Research, Mayo Clinic, Rochester, MN 55905, USA.; 6 Cancer Genomics Research Laboratory, Division of Cancer Epidemiology and Genetics, National Cancer Institute, Gaithersburg, MD 20877, USA.; 7 Basic Science Program, Frederick National Laboratory for Cancer Research in the Laboratory of Integrative Cancer Immunology, National Cancer Institute, Bethesda, MD 20892, USA.; 8 Ragon Institute of MGH, Cambridge, MA 02139, USA.; 9 Cancer Epidemiology Division, Cancer Council Victoria, Melbourne, Victoria 3004, Australia.; 10 Centre for Epidemiology and Biostatistics, School of Population and Global Health, The University of Melbourne, Melbourne, Victoria 3010, Australia.; 11 Precision Medicine, School of Clinical Sciences at Monash Health, Monash University, Clayton, Victoria 3800, Australia.; 12 Channing Division of Network Medicine, Department of Medicine, Brigham and Women's Hospital and Harvard Medical School, Boston, MA 02115, USA.; 13 Department of Medical Epidemiology and Biostatistics, Karolinska Institutet, Stockholm 17176, Sweden.; 14 Department of Epidemiology, Harvard T.H. Chan School of Public Health, Boston, MA 02115, USA.; 15 Institute of Health and Society, Clinical Effectiveness Research Group, University of Oslo, Oslo 0315, Norway.; 16 Department of Obstetrics and Gynecology, New York University School of Medicine, New York, NY 10016, USA.; 17 Department of Population Health, New York University School of Medicine, New York, NY 10016, USA.; 18 Perlmutter Comprehensive Cancer Center, NYU Langone Health, New York, NY 10016, USA.; 19 Division of Cancer Epidemiology, German Cancer Research Center (DKFZ), Heidelberg, Baden-Württemberg 69120, Germany.; 20 Cancer Epidemiology Research Programme, Catalan Institute of Oncology-IDIBELL, L'Hospitalet de Llobregat, Barcelona 08908, Spain.; 21 Consortium for Biomedical Research in Epidemiology and Public Health (CIBERESP), Barcelona 08036, Spain.; 22 Regional Cancer Prevention Laboratory, Institute for Cancer Research, Prevention and Clinical Network (ISPRO), Florence 50139, Italy.; 23 Stony Brook Cancer Center, Stony Brook University, Stony Brook, NY 11794, USA.; 24 Department of Medical and Surgical Sciences, University of Bologna, Bologna 41026, Italy.; 25 Department of Epidemiology and Biostatistics, University of California San Francisco, San Francisco, CA 94118, USA.; 26 International Agency for Research on Cancer (IARC), Lyon 69372, France.; 27 Genome Sciences Centre, BC Cancer Agency, Vancouver, British Columbia V5Z1L3, Canada.; 28 Department of Biomedical Physiology and Kinesiology, Simon Fraser University, Burnaby, British Columbia V5A1S6, Canada.; 29 Genomic Epidemiology Group, German Cancer Research Center (DKFZ), Heidelberg 69120, Germany.; 30 Center of Research in Epidemiology and Statistics Sorbonne Paris Cité (CRESS), UMR1153, INSERM, Villejuif 75004, France.; 31 Department of Public Health, Clinical and Molecular Medicine, University of Cagliari, Monserrato, Cagliari 09042, Italy.; 32 Bill Lyons Informatics Centre, UCL Cancer Institute, University College London, London WC1E 6DD, UK.; 33 INSERM U1052, Cancer Research Center of Lyon, Centre Léon Bérard, Lyon 69008, France.; 34 Department of Preventive Medicine, USC Keck School of Medicine, University of Southern California, Los Angeles, CA 90033, USA.; 35 Norris Comprehensive Cancer Center, USC Keck School of Medicine, University of Southern California, Los Angeles, CA 90033, USA.; 36 Department of Internal Medicine and Huntsman Cancer Institute, University of Utah School of Medicine, Salt Lake City, UT 84112, USA.; 37 Department of Cancer Epidemiology and Genetics, Masaryk Memorial Cancer Institute, Brno 656 53, Czech Republic.; 38 Department of Population Science, American Cancer Society, Atlanta, GA 30303, USA.; 39 Department of Hematology, Centre Léon Bérard, Lyon 69008, France.; 40 INSERM U1052, Cancer Research Center of Lyon, Lyon-1 University, Pierre-Bénite Cedex 69008, France.; 41 Department of Immunology, Genetics and Pathology, Uppsala University, Uppsala 75105, Sweden.; 42 Department of Internal Medicine, Mayo Clinic, Rochester, MN 55905, USA.; 43 Department of Epidemiology Research, Division of Health Surveillance and Research, Statens Serum Institut, Copenhagen 2300, Denmark.; 44 Division of Endocrinology, Diabetes and Metabolism, The Ohio State University, Columbus, OH 43210, USA.; 45 Department of Internal Medicine, Carver College of Medicine, The University of Iowa, Iowa City, IA 52242, USA.; 46 U1231, Registre des Hémopathies Malignes de Côte d’Or, University of Burgundy and Dijon University Hospital, Dijon 21070, France.; 47 Department of Medicine, Stanford University School of Medicine, Stanford, CA 94305, USA.; 48 Environmental and Occupational Epidemiology Branch-Cancer Risk Factors and Lifestyle Epidemiology Unit, Institute for Cancer Research, Prevention and Clinical Network (ISPRO), Florence 50139, Italy.; 49 Department of Pathology, AP-HP, Necker Enfants Malades, Université Paris Descartes, EA 7324, Sorbonne Paris Cité 75015, France.; 50 Registre des Hémopathies Malignes de la Gironde, Institut Bergonié, Bordeaux Cedex 33076, France.; 51 Center for Chronic Immunodeficiency, University Medical Center Freiburg, Freiburg, Baden-Württemberg 79108, Germany.; 52 Department of Epidemiology, University of North Carolina at Chapel Hill, Chapel Hill, NC 27599, USA.; 53 Carolina Center for Genome Sciences, University of North Carolina at Chapel Hill, Chapel Hill, NC 27599, USA.; 54 Department of Medicine, Memorial Sloan Kettering Cancer Center, New York, NY 10065, USA.; 55 School of Public Health, Imperial College London, London W2 1PG, UK.; 56 Department of Hematology, Hospices Civils de Lyon, Pierre Benite Cedex 69495, France.; 57 Department of Hematology, Université Lyon-1, Pierre Benite Cedex 69495, France.; 58 Department of Family Medicine and Public Health Sciences, Wayne State University, Detroit, MI 48201, USA.; 59 Department of Hematology and Medical Oncology, Emory University School of Medicine, Atlanta, GA 30322, USA.; 60 Department of Medicine, Solna, Karolinska Institutet, Stockholm 17176, Sweden.; 61 Hematology Center, Karolinska University Hospital, Stockholm 17176, Sweden.; 62 Genetic Epidemiology Laboratory, Department of Pathology, University of Melbourne, Melbourne, Victoria 3010, Australia.; 63 Cancer Control Research, BC Cancer Agency, Vancouver, British Columbia V5Z1L3, Canada.; 64 School of Population and Public Health, University of British Columbia, Vancouver, British Columbia V6T1Z3, Canada.; 65 School of Nursing, Psychotherapy and Community Health, Dublin City University, Dublin 9, Ireland.; 66 Division of Public Health Sciences, Fred Hutchinson Cancer Research Center, Seattle, WA 98117, USA.; 67 Cancer Epidemiology Unit, University of Oxford, Oxford OX3 7LF, UK.; 68 Centre for Big Data Research in Health, University of New South Wales, Sydney, New South Wales 2052, Australia.; 69 Institute for Risk Assessment Sciences, Utrecht University, Utrecht 3584 CG, The Netherlands.; 70 Julius Center for Health Sciences and Primary Care, University Medical Center Utrecht, Utrecht 3584 CX, The Netherlands.; 71 Concord Clinical School, University of Sydney, Concord, New South Wales 2139, Australia.; 72 Department of Environmental Health Sciences, Yale School of Public Health, New Haven, CT 06520, USA.; 73 Department of Epidemiology, Brown University, Providence, RI 02903, USA.

**Keywords:** Non-Hodgkin lymphoma, homozygosity, chronic lymphocytic leukemia, follicular lymphoma, diffuse large B-cell lymphoma, marginal zone lymphoma

## Abstract

**Aim:**

Recessive genetic variation is thought to play a role in non-Hodgkin lymphoma (NHL) etiology. Runs of homozygosity (ROH), defined based on long, continuous segments of homozygous SNPs, can be used to estimate both measured and unmeasured recessive genetic variation. We sought to examine genome-wide homozygosity and NHL risk.

**Methods:**

We used data from eight genome-wide association studies of four common NHL subtypes: 3061 chronic lymphocytic leukemia (CLL), 3814 diffuse large B-cell lymphoma (DLBCL), 2784 follicular lymphoma (FL), and 808 marginal zone lymphoma (MZL) cases, as well as 9374 controls. We examined the effect of homozygous variation on risk by: (1) estimating the fraction of the autosome containing runs of homozygosity (FROH); (2) calculating an inbreeding coefficient derived from the correlation among uniting gametes (F3); and (3) examining specific autosomal regions containing ROH. For each, we calculated beta coefficients and standard errors using logistic regression and combined estimates across studies using random-effects meta-analysis.

**Results:**

We discovered positive associations between FROH and CLL (β = 21.1, SE = 4.41, *P* = 1.6 × 10^−6^) and FL (β = 11.4, SE = 5.82, *P* = 0.02) but not DLBCL (*P* = 1.0) or MZL (*P* = 0.91). For F3, we observed an association with CLL (β = 27.5, SE = 6.51, *P* = 2.4 × 10^−5^). We did not find evidence of associations with specific ROH, suggesting that the associations observed with FROH and F3 for CLL and FL risk were not driven by a single region of homozygosity.

**Conclusion:**

Our findings support the role of recessive genetic variation in the etiology of CLL and FL; additional research is needed to identify the specific loci associated with NHL risk.

## INTRODUCTION

Disentangling the heritable component of non-Hodgkin lymphoma (NHL) and its subtypes is an active area of research. An early study of familial aggregation in NHL reported an increased risk of NHL among siblings, but not parents or offspring, of an index NHL case^[1]^. Several subsequent studies found an elevated risk of NHL associated with a first-degree family history of NHL with the highest risks for siblings^[2,3]^. Studies have also reported higher NHL subtype-specific risks for first-degree relatives of cases affected with a given NHL subtype^[4,5]^, suggesting a degree of subtype specificity. In general, these findings suggest genetic factors are important in NHL etiology and, in particular, the potential role of recessively acting genetic risk alleles, but they also underscore the potential for genetic heterogeneity in susceptibility to different NHL subtypes. Part of the difficulty in characterizing risk and inheritance patterns of NHL subtypes is the limited study sample size, especially when examining specific subtypes. Genome-wide association studies (GWAS) have identified multiple susceptibility loci associated with four major subtypes of NHL^[6-11]^, but a substantial fraction of the disease heritability remains unexplained. Most GWAS performed assume an additive model of genetic risk, which has statistical power to detect allelic associations acting through a variety of mechanisms but may not efficiently detect recessive effects, particularly as minor allele frequency and imputation quality decrease^[12]^. Therefore, recessively acting loci, particularly those with low minor allele frequency, could be missed by current genome-wide scans and represent potential novel disease-associated loci.

The widespread use of dense genotyping arrays has led to the identification of sizeable genomic regions consisting of consecutive homozygous SNPs in non-consanguineous populations^[13]^. These runs of homozygosity (ROH) vary in length, with short ROH persisting from ancient relatedness and long ROH of several megabases arising from recent parental relatedness^[14]^. The use of ROH as a measure of the burden of homozygosity has been demonstrated to perform better at identifying rare, recessive mutations than a conventional SNP-by-SNP analysis^[15]^. Furthermore, studies incorporating whole-exome sequencing have uncovered an enrichment of deleterious variants in ROH^[16]^. In recent years, studies have examined the association between ROH and various cancers^[17-21]^, among other complex common diseases and traits^[22]^. Although no association was observed with the cumulative distribution of ROH, individual ROH were associated with the risk of childhood acute lymphoblastic leukemia^[19]^. Hodgkin lymphoma has been inconsistently associated with specific ROH and overall homozygosity^[20,21]^. To our knowledge, no studies have examined ROH in association with adult NHL.

The goal of the present study was to investigate the association of homozygosity with the risk of four major NHL subtypes: chronic lymphocytic leukemia/small chronic lymphocytic leukemia (CLL), diffuse large B-cell lymphoma (DLBCL), follicular lymphoma (FL), and marginal zone lymphoma (MZL). Several measures of homozygosity were tested against NHL risk using data from eight GWAS.

## METHODS

We used data from eight previous GWAS of NHL^[6,8,10,11]^ composed of cases and controls of European ancestry [Supplementary Table 1 and 2]. The National Cancer Institute (NCI) NHL GWAS included cases with one of four common NHL subtypes and controls from 22 studies of NHL: 9 prospective cohort studies, 8 population-based case-control studies, and 5 hospital- or clinic-based case-control studies or case series. These 22 studies comprising the NCI NHL GWAS were genotyped using the Illumina OmniExpress or Omni2.5 arrays and analyzed as a single study. The other seven GWAS were the University of California at San Francisco Molecular Epidemiology of Non-Hodgkin Lymphoma study (UCSF2)^[23]^, the University of California at San Francisco Molecular Epidemiology of Non-Hodgkin Lymphoma study (UCSF1) combined with controls from the Nurses’ Health Study (NHS)^[24]^, the Scandinavian Lymphoma Etiology Study (SCALE)^[25]^, the Groupe d’Etude des Lymphomes de l’Adulte (GELA) study combined with controls from the European Prospective Investigation into Cancer, Chronic Diseases, Nutrition, and Lifestyles (EPIC), the Mayo Clinic Case-Control Study of Diffuse Large B-cell Lymphoma (Mayo), the Genetic Epidemiology of CLL Consortium (GEC), and the Utah Chronic Lymphocytic Leukemia Study (Utah). Genotyping was performed on commercially available Illumina and Affymetrix platforms [Supplementary Table 1]. Details, including information on quality control and data cleaning, have been previously reported^[6,8,10,11]^. All studies obtained informed consent from participants and were approved by their appropriate Institutional Review Boards.

Prior to analysis, additional quality control and filtering were applied to each GWAS separately, including removal of SNPs with a minor allele frequency < 0.05, > 3% missing, or Hardy-Weinberg *P*-value < 1 × 10^−6^ among controls, and removal of subjects with call rates < 97%. After quality control metrics, genotype data were available for 10,467 NHL cases, including 3061 CLL, 3814 DLBCL, 2784 FL, and 808 MZL cases, as well as 9374 controls [Supplementary Table 2].

We used PLINK1.9^[26,27]^ to identify ROH; specifically, we used the two-step command *--homozyg.* In the first step, PLINK1.9 identifies directly genotyped SNPs that are possibly within an ROH by looking at 50-SNP sliding windows across the genome and flagging all SNPs that are encompassed by at least 5% of fully homozygous windows. For this step, we allowed one heterozygous SNP and up to five SNPs with no calls within each window to account for a small amount of possible genotyping error and loss. In the second step, ROH are identified from these sliding windows by requiring a minimum number of consecutive homozygous SNPs. We required at least 100 consecutive homozygous SNPs for each ROH and that these SNPs span at least 1500 kilobases (kb), with at least one SNP every 50 kb and the maximum gap between SNPs of 5000 kb. These parameters were selected with reference to the “ROH_1.5Mb” ROH calling parameters used by Gazal *et al.*^[28]^ We restricted analyses to the autosomal chromosomes.

To estimate the extent of homozygosity across the genome, we calculated the fraction of the autosome covered by ROH (FROH) by summing the lengths of ROH and dividing by 3 × 10^9^ base pairs as the approximate size of the autosome for all GWAS. As another measure to assess homozygosity, we also quantified and tested differences in relatedness across the genome in our study using a variant of the inbreeding coefficient, F3^[29]^. F3, which estimates the correlation between uniting gametes, is an alternative to ROH-based estimates with potentially reduced bias and standard errors^[30]^. We estimated F3 using the *-ibc* command in PLINK1.9. To estimate the association of FROH and F3 with NHL, we then estimated beta coefficients and standard errors for each GWAS using logistic regression, adjusting for age, sex (except in the UCSF1/NHS study, where all controls were female), fraction of missing SNPs, and the ten principal components of ancestry to account for population stratification. The fraction of missing SNPs was calculated for each participant as the number of SNPs without calls divided by the total number of SNPs genotyped on the array that passed quality control metrics. Associations were combined across GWAS for each subtype of NHL using random-effects meta-analysis implemented with the command “*metan*” in STATA v15.

After determining ROH as described above, we also tested whether specific genomic regions encompassed by ROH were associated with risk of each of the four NHL subtypes. We divided each autosomal chromosome into “bins” of 500 kb in length. We then calculated the midpoint of each identified ROH and assigned it to the corresponding bin. Each study participant in the analysis was therefore categorized as either homozygous (exposed) or heterozygous (unexposed) at each bin across the autosome. We calculated beta coefficients and standard errors for the association between presence of an ROH in each bin and risk of NHL subtype within each GWAS using logistic regression, adjusting for age, sex (except in the UCSF1/NHS study), fraction of missing SNPs, and ten principal components of ancestry. Results were combined across GWAS using METAL^[31]^, and multiple-testing adjustment was performed using a Bonferroni correction.

## RESULTS

Table 1 presents summary statistics for the ROH, FROH, and F3 by GWAS. Among participants, the median total length of ROH ranged from 11,535 to 23,014 kb depending on the GWAS. The median number of ROH per individual ranged from 4 in the UCSF2 GWAS, which used an older and less dense GWAS chip, to 8 in the GEC GWAS, which included familial CLL cases. Median FROH ranged from 0.38% to 0.77% of the autosome. Median F3 ranged from −6.99 × 10^−4^ to 3.02 × 10^−3^ [Table 1].

We discovered a positive association between the risk of CLL and increased homozygosity as measured by FROH (β = 21.1, 95% SE = 4.41, *P* = 1.6 × 10^−6^) and F3 (β = 27.5, SE = 6.51, *P* = 2.4 × 10^−5^) [Table 2] with limited evidence of between-study heterogeneity (*P*_het_ = 0.42 and 0.11, respectively) [Supplementary Table 3]. As CLL is an indolent lymphoma and there is a potential for tumor DNA contamination in the blood drawn for genotyping, we performed a sensitivity analysis using only CLL cases and controls from prospective nested case-control studies (ATBC, CPS-II, EPIC, HPFS, MCCS, NHS, NYU-WHS, PLCO, and WHI) from the NCI NHL GWAS, where the DNA was often collected many years prior to diagnosis. Despite the reduction in the number of CLL cases (*n* = 2140 to 889), the estimated association parameters for FROH and F3 were similar (FROH: β = 21.3, SE = 7.76, *P* = 6.04 × 10^−3^; F3: β = 22.5, SE = 5.45, *P* = 3.57 × 10^−5^).

**Figure 1.**
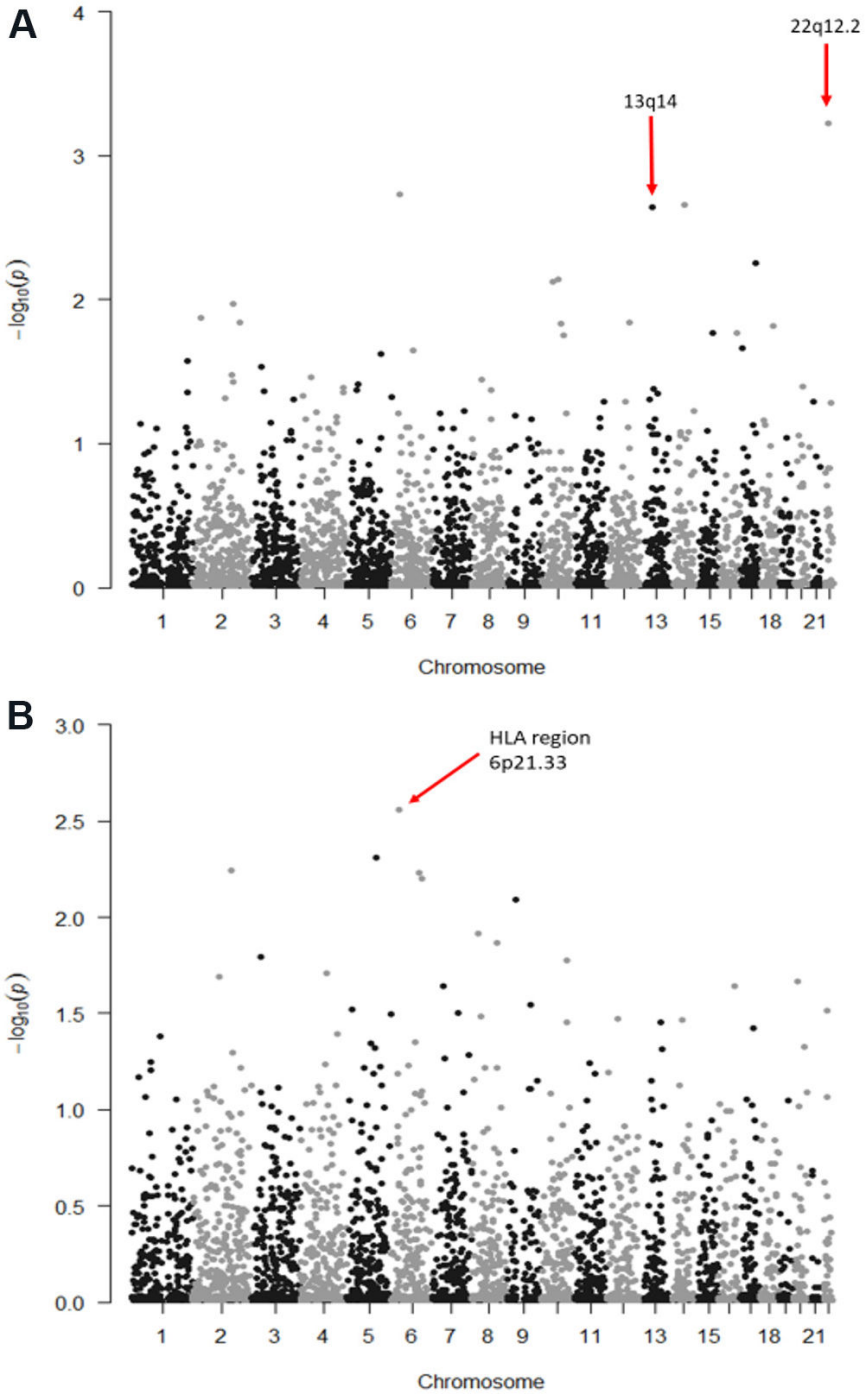
Manhattan plots of log_10_(*P*) values for the association between runs of homozygosity (ROH) and the risk of chronic lymphocytic leukemia (CLL) (A) and follicular lymphoma (FL) (B). Here, ROH were divided into 500-kb bins across each chromosome, and each bin was tested for its association with CLL or FL. No bins reached statistical significance after correction for multiple testing.

Figure 1A shows the *P*-values (−log_10_) from the meta-analysis assessing the associations of CLL with ROH centered in each 500 kb bin in the autosome. No bins reached statistical significance after correction for multiple testing (CLL Bonferroni alpha level = 0.05/45,590 bins = 1.1 × 10^−5^); the most significantly associated bin was located at chromosome 22q12.2 (*P* = 5.92 × 10^−4^). However, one of the top ten associated bins overlapped with the chromosome 13q14 region, a region where a somatic deletion is often seen in CLL^[32]^. To test whether the association between FROH and CLL was due to mosaicism at 13q14, we excluded the 45 CLL cases identified to have this deletion from the NCI NHL GWAS. After removal of these 45 cases, the association between FROH and CLL was slightly attenuated but remained statistically significant (β = 16.34, SE = 4.74, *P* = 5.71 × 10^−4^), similar to the results from the entire NCI NHL GWAS for CLL (β = 18.9, SE = 4.76, *P* = 6.73 × 10^−5^).

As structural alterations in chromosomes, such as trisomy 12, are a hallmark of CLL and could potentially be mistakenly called as ROH using this method, we conducted a sensitivity analysis excluding chromosomes 12 and 13, which are frequently altered in CLL, from the meta-analysis^[33]^. After excluding these two chromosomes, the results for the association between FROH and CLL were found to be similar (β = 19.3, SE = 4.4, *P* = 9.0 × 10^−6^) to previous results for all 22 autosomal chromosomes. Similarly, for F3, exclusion of chromosomes 12 and 13 led to results that are similar in magnitude and remain statistically significant (β = 17.6, SE = 7.4, *P* = 0.02). Between-study heterogeneity (*I*^2^ = 62.2%, *P*_het_ = 0.05) was detected in this sensitivity analysis, largely attributable to the GEC GWAS.

We also identified positive associations between FL risk and increased FROH (β = 11.4, SE = 5.82, *P* = 0.02) but not F3 (β = 13.2, SE = 8.01, *P* = 0.10) [Table 2]. There was evidence of between-study heterogeneity in the meta-analysis of F3 and FL (*I*^2^ = 64.2%, *P* = 0.04) due to the USCF1/NHS GWAS, but not in the meta-analysis of FROH and FL (*I*^2^ = 5.3%, *P* = 0.37) [Supplementary Table 3]. We performed a sensitivity analysis using only cases and controls collected from prospective nested case-control studies (ATBC, CPS-II, EPIC, HPFS, MCCS, NHS, NYU-WHS, PLCO, and WHI) as part of the NCI NHL GWAS. This resulted in a reduction of the number of cases (*n* = 2085 to 521) and statistical power, but the association between FROH and risk of FL was qualitatively similar (β = 14.1, SE = 11.4, *P* = 0.21). Given the importance of the HLA region in FL risk, we conducted a sensitivity analysis removing chromosome 6 from estimations of FROH and F3 and repeating the meta-analysis of the four GWAS. The results for both FROH (β = 12.5, SE = 6.29, *P* = 0.047) and F3 (β = 11.1, SE = 9.26, *P* = 0.23) were found to be similar to the between-study heterogeneity observed for F3 (*I*^2^ = 73.1%, *P* = 0.01) due to the UCSF1/NHS GWAS.

Figure 1B shows the *P*-values (−log_10_) from the meta-analysis of logistic regression models estimating the associations of FL with ROH centered in each 500-kb bin in the autosome. No bins reached statistical significance assuming a Bonferroni-corrected alpha level. The most significant association was for a bin overlapping the HLA region 6p21.33 (*P* = 2.78 × 10^−3^).

No associations were observed with either FROH or F3 and the risk of DLBCL or MZL [Table 2]; however, between-study heterogeneity in DLBCL risk was present for both FROH (*I*^2^ = 72.5%, *P*_het_ = 0.01) and F3 (*I*^2^ = 82.4%, *P*_het_ = 0.001) [Supplementary Table 3]. In a leave-one-out sensitivity analysis [Supplementary Table 4], removal of either the NCI NHL GWAS or the GELA/EPIC GWAS, which had association results in opposite directions, reduced the *I*^2^ among the remaining three GWAS to 0%. Without the GELA/EPIC GWAS, there were significant associations between FROH and DLBCL (β = 13.1, SE = 4.62, *P* = 0.004) and between F3 and DLBCL (β = 13.4, SE = 3.18, *P* = 2.4 × 10^−5^).

## DISCUSSION

To our knowledge, the present study represents the first analysis of the association between homozygosity and risk of four major NHL subtypes. We assessed genome-wide homozygosity using F3 and FROH, where the beta coefficient represents the log odds for a one-unit change in the fraction of ROH. We discovered that increased genome-wide homozygosity was associated with an increased risk of CLL and FL. We estimated that a 0.1% increase in the fraction of ROH was associated with a 2% increase in CLL risk and 1% increase in FL risk compared to those without any ROH. While we did not identify specific regions containing novel associations with these four subtypes of NHL, our analyses suggest that recessive genetic variation is likely to play a role in the risk of these two NHL subtypes and may account for a fraction of the unexplained heritability of these diseases. Our study did not find convincing evidence that recessive variation is a significant contributor to the risk of DLBCL and MZL, further supporting the hypothesis that each NHL subtype is likely to have its own genetic architecture of disease susceptibility.

Evidence suggests that CLL has high heritability with first-degree relatives having a 6-8.5-fold increase in risk of CLL^[5,34]^. Approximately 17%-25% of the familial relative risk of CLL is explained by currently known risk loci^[6,9]^, leaving a sizable fraction that remains undiscovered. We discovered strong associations between CLL and genome-wide homozygosity as measured by FROH and F3. These findings suggest that recessive genetic variants are likely to contribute to risk.

CLL is a hematologic malignancy characterized by large-scale chromosomal alterations^[35]^ and is often preceded by a long-lasting pre-malignant stage of monoclonal B-cell lymphocytosis (MBL)^[36]^. It is possible that cases with diagnosed CLL at the time of enrollment or those with unidentified MBL had tumor DNA in their blood samples, and that some mosaic events may have been erroneously picked up as ROH or homozygous genotypes due to the calling method utilized. This could have inflated our association results, but our sensitivity analyses suggested that any influence of mosaicism on our findings was likely to be small. We excluded CLL cases with DNA collected after diagnosis in the NCI NHL GWAS and saw similar results to our primary analysis, suggesting that tumor contamination is unlikely to be responsible for the observed association. Further sensitivity analysis excluding individuals with known 13q14 deletion in NCI NHL GWAS showed that the associations between FROH and F3 and CLL were only slightly attenuated. We also removed chromosomes 12 and 13 from the calculation of FROH and F3, as these two chromosomes are frequently altered in newly diagnosed CLL^[33]^, and again saw results similar to the primary analysis. While we cannot exclude the possibility that some of the association could be due to erroneous homozygosity calling of structural variation, the robustness of the associations indicates that potential bias from these sources probably does not explain the observed association.

We did not identify any specific homozygous regions that were associated with CLL risk after adjustment for multiple comparisons, but we observed nominal associations with ROH at chromosomes 22q12.2 and 13q14.2. The chromosome 22q12.2 region includes POZ/BTB and AT hook containing zinc finger 1 (*PATZ1*), which is a transcription factor that interacts with p53 and may modulate p53-mediated apoptosis^[37]^. The region also contains phosphoinositide-3-kinase interacting protein 1 (*PIK3IP1*), which inhibits antitumor immunity^[38]^. The chromosome 13q14.2 region contains several genes of potential interest, including RB transcriptional corepressor 1 (*RB1*) and cysteinyl-leukotriene receptor 2 (*CYSLTR2*). RB1 is a known tumor suppressor, which is frequently somatically deleted in CLL^[32]^. CYSLTR2 is part of the leukotriene pathway and thought to play a role in lymphocyte proliferation and maturation^[39]^.

For FL, we discovered a positive association between FROH and risk of FL, suggesting recessive genetic variation is a contributor to risk. Previous epidemiologic work has shown that family history of NHL is a risk factor for FL^[40]^, and first-degree relatives have a 4-6-fold increase risk of FL^[4,5]^. The largest GWAS of FL to date detected multiple genetic loci associated with risk, with the strongest associations seen in the HLA region^[10]^. Consistent with our findings, a previous study of first-degree relatives suggested that FL may follow an autosomal recessive mode of inheritance^[5]^. In sensitivity analyses, we showed that results for FROH are similar after excluding the entirety of chromosome 6, suggesting that our findings were not due to the HLA region. This finding supports the role of non-HLA variation in the etiology of FL and provides evidence that there may be additional rare, recessive loci that are associated with the risk of FL. We observed similar results after limiting to cases with prospectively collected DNA, suggesting that tumor contamination is unlikely. Although we did not find any individual regions (based on 500-kb bins of ROH) that were significantly associated with the risk of FL after adjustment for multiple testing, we observed nominal evidence for homozygosity overlapping the HLA region at chromosome 6p21.33. We previously reported that homozygosity for classical HLA class II alleles, such as *HLA-DRB1*, was associated with an increased risk of FL compared to individuals with heterozygosity^[41]^. These findings are consistent with the hypothesis that HLA homozygosity may increase risk by reducing an individual’s ability to recognize a diverse set of foreign antigens.

DLBCL shows a strong association with family history of NHL in epidemiologic studies^[42]^, and it has been previously estimated that 16% of the variation in DLBCL is due to common genetic variants^[8]^. Overall, we did not observe an association between DLBCL and homozygosity, suggesting the recessive variation may not have a strong role in DLBCL susceptibility, a finding consistent with a previous study of DLBCL among first-degree relatives^[5]^. The meta-analysis for DLBCL was affected by substantial between-study heterogeneity (*P*_het_ = 0.01 for FROH and *P*_het_ = 0.001 for F3). In the leave-one-out meta-analysis, removing either the NCI NHL GWAS or the GELA/EPIC GWAS reduced the *I*^2^ among the remaining three GWAS to 0%, and significant associations were observed with FROH and F3 after removing GELA/EPIC. The GELA/EPIC GWAS was different from the other studies in that it included patients from clinical trials, who were slightly younger and less likely to be female than the other studies [Supplementary Table 2]. In addition, this GWAS combined cases from French clinical trials with controls from various European countries in the EPIC cohort. It is possible that the degree of population matching, while adequate for a GWAS, is not sufficient for analyses of F3 and FROH, which are known to be especially sensitive to population stratification^[43]^. Even after adjustment using principal components, some population substructure may have affected the GELA/EPIC study, leading to results that differ from the other DLBCL studies. Our analysis of FL similarly included one GWAS that combined the cases and controls from 2 different United States studies. Although we did observe some heterogeneity among the FL studies for F3, we did not observe such heterogeneity for FROH and FL, suggesting the F3 may be more sensitive to population substructure than FROH. The patients in the UCSF1/NHS study were slightly younger and less likely to be female than the cases in the NCI GWAS, which could have also contributed to the heterogeneity in results.

DLBCL is known to have substantial disease heterogeneity^[44,45]^. It is possible that a greater understanding of the heterogeneity of DLBCL and a further molecular subtype-specific analysis, which was not possible for the present study, may allow a better elucidation of any association between ROH and disease risk. While the analysis of F3 and DLBCL was similarly affected by between-study heterogeneity [Supplementary Table 3], a potential advantage of F3 over FROH as a measure of homozygosity is its expected smaller variance and bias^[30]^. In addition, the method of calculating FROH used in this study does not count homozygosity below a length threshold of 1500 kb^[28]^. It is possible that a portion of the recessive genetic variation in DLBCL resides in short, common ROH of ancient ancestry^[14]^ that was unmeasured by our approach.

Although family history of NHL or other hematologic cancers has been associated with increased risk of MZL^[46]^, we did not find evidence linking FROH or F3 to MZL. Our published GWAS of MZL found two independent loci conferring risk, both in the HLA region of the genome^[11]^, and a follow-up study reported that homozygosity at class I HLA-B and -C and class II HLA-DRB1 loci was associated with increased risk of MZL^[41]^. Although our study did not detect an association with homozygosity more broadly across the genome, local homozygosity, at least in select regions, is still likely to play a role in risk. Of the four subtypes of NHL examined in the present study, MZL is the least common. Our power to detect an association with MZL was limited due to the small sample size, which was possibly compounded by the known heterogeneity within MZL, with multiple recognized subtypes^[47]^. Given that we expect ROH to capture the effects of small, scattered, recessive genotypes, a larger sample size is likely needed to elucidate the genetic etiology of MZL. As other highly heritable traits, such as certain autoimmune diseases and atopy, are associated with the risk of MZL^[46]^, identifying shared genetic architecture with these more common phenotypes may be a complementary strategy to further elucidate the underlying genetic architecture.

This study had both limitations and strengths. The use of FROH as a measure of homozygosity is known to require large sample sizes^[30]^. We were able to combine eight GWAS across four subtypes of NHL to increase our sample size and provide a fairly comprehensive analysis of recessive inheritance. However, as discussed, we may have had insufficient sample size to detect associations for MZL. Further, we could not separately examine clinically relevant molecular subtypes of DLBCL. ROH patterns are exquisitely sensitive to population history^[48]^. Thus, we restricted our study population to individuals of European ancestry and adjusted for principal components of ancestry. This limited the potential for problematic population stratification in our study sample, but it also reduced the generalizability of the findings to individuals of other ancestries. To date, the optimal approach to analyze specific ROH to identify genomic regions with novel, recessively acting risk alleles has not been established, but both the ROH calling procedure and the use of F3 are accepted as means of identifying recent relatedness in outbred European populations^[28,30]^ and can provide evidence for the presence of recessive loci. Although it is possible that the associations between FROH and F3 and CLL are due in part to DNA contamination by tumor cells, our sensitivity analyses suggest that germline homozygosity is likely an independent risk factor for CLL. Finally, although our study provides clues as to the genetic etiology of NHL, the clinical value of these findings is uncertain. Additional studies are needed to further elucidate the role of recessive variation in NHL risk.

In conclusion, we provide new evidence for the role of recessive genetic variation in the risk of CLL and FL in outbred European-ancestry populations. The knowledge that recessive variation in disease susceptibility to NHL is likely to be present suggests that further studies should be undertaken to identify the specific loci responsible for the associations reported here. As GWAS increase in sample size, they will have greater statistical power to identify recessive genetic variants-associated risk and further characterize the underlying genetic architecture of specific NHL subtypes.

## Supplementary Material

Supplementary Tables

## Figures and Tables

**Table 1.** Summary of genome-wide homozygosity measures by GWAS and case-control status*

Subtype	Study	No. cases	No. controls	Median ROH total length (kb)	IQR ROH total length (kb)	Median number of ROH	IQR number of ROH	Median FROH	IQR FROH	Median F3	IQR F3
CLL	NCI	Cases	2140	18593	13057-25486	7	5-9	0.62%	0.44%-0.79%	2.57 × 10^−3^	−1.34 × 10^−3^−7.49 × 10^−3^
		Controls	6105	18045	12674-24396	7	5-9	0.60%	0.42%-0.81%	1.48 × 10^−3^	−2.07 × 10^−3^−5.36 × 10^−3^
	GEC	Cases	387	23014	16496-29054	8	6-10	0.77%	0.55%-0.97%	6.41 × 10^−4^	−3.66 × 10^−3^−4.85 × 10^−3^
		Controls	294	21920	14852-28391	8	6-10	0.73%	0.50%-0.95%	−6.99 × 10^−4^	−4.90 × 10^−3^−3.06 × 10^−3^
	UCSF2	Cases	213	12993	7893-19530	4	3-6	0.43%	0.26%-0.65%	2.73 × 10^−3^	−0.63 × 10^−3^−8.83 × 10^−3^
		Controls	746	12173	7523-17111	4	3-6	0.41%	0.25%-0.57%	1.51 × 10^−3^	−2.25 × 10^−3^−5.72 × 10^−3^
	Utah	Cases	321	18806	13535-24431	7	5-9	0.63%	0.45%-0.81%	3.69 × 10^−4^	−2.79 × 10^−3^−4.71 × 10^−3^
		Controls	403	17452	12288-22866	6	5-8	0.58%	0.41%-0.76%	9.47 × 10^−4^	−2.09 × 10^−3^−3.89 × 10^−3^
DLBCL	NCI	Cases	2621	18046	12671-24990	7	5-9	0.60%	0.42%-0.83%	2.11 × 10^−3^	−1.48 × 10^−3^−6.54 × 10^−3^
		Controls	6105	18199	12753-24519	7	5-9	0.61%	0.43%-0.82%	1.46 × 10^−3^	−2.05 × 10^−3^−5.39 × 10^−3^
	Mayo	Cases	393	16586	12167-21774	6	5-8	0.55%	0.41%-0.73%	1.67 × 10^−4^	−3.03 × 10^−3^−3.07 × 10^−3^
		Controls	172	16451	11368-23319	6	4-8	0.55%	0.38%-0.78%	2.19 × 10^−4^	−2.55 × 10^−3^−2.94 × 10^−3^
	UCSF2	Cases	253	11535	5957-19153	4	3-6	0.38%	0.20%-0.64%	1.69 × 10^−3^	−1.62 × 10^−3^−6.60 × 10^−3^
		Controls	745	12182	7554-17158	4	3-6	0.41%	0.25%-0.57%	1.48 × 10^−3^	−2.20 × 10^−3^−5.82 × 10^−3^
	GELA/EPIC	Cases	547	15526	10391-21466	6	4-7	0.52%	0.35%-0.72%	1.27 × 10^−3^	−2.27 × 10^−3^−5.00 × 10^−3^
		Controls	525	17327	11782-23437	6	4-8	0.58%	0.39%-0.78%	2.56 × 10^−3^	−0.87 × 10^−3^−6.14 × 10^−3^
FL	NCI	Cases	2085	17838	12387-24319	7	5-9	0.59%	0.41%-0.81%	1.41 × 10^−3^	−2.14 × 10^−3^−6.15 × 10^−3^
		Controls	6105	18110	12753-24512	7	5-9	0.60%	0.43%-0.82%	1.49 × 10^−3^	−2.06 × 10^−3^−5.41 × 10^−3^
	UCSF1/NHS	Cases	119	17058	12645-22150	7	5-8	0.57%	0.42%-0.74%	8.51 × 10^−5^	−3.57 × 10^−3^−3.05 × 10^−3^
		Controls	340	17144	12100-24513	6.5	5-9	0.57%	0.40%-0.82%	6.35 × 10^−4^	−3.05 × 10^−3^−4.29 × 10^−3^
	UCSF2	Cases	209	13621	8911-19655	5	3-7	0.45%	0.30%-0.66%	3.02 × 10^−3^	−1.34 × 10^−3^−7.58 × 10^−3^
		Controls	745	12071	7517-17115	4	3-6	0.40%	0.25%-0.57%	1.50 × 10^−3^	−2.16 × 10^−3^−5.76 × 10^−3^
	SCALE	Cases	371	12582	7643-19776	5	3-7	0.42%	0.25%-0.66%	3.02 × 10^−3^	−0.50 × 10^−3^−7.52 × 10^−3^
		Controls	790	12132	7506-17654	5	3-6	0.40%	0.25%-0.59%	1.22 × 10^−3^	−1.60 × 10^−3^−5.00 × 10^−3^
MZL	NCI	Cases	808	18097	12831-24555	7	5-9	0.60%	0.43%-0.82%	1.83 × 10^−3^	−1.54 × 10^−3^−7.30 × 10^−3^
		Controls	6102	18268	12811-24623	7	5-9	0.61%	0.43%-0.82%	1.46 × 10^−3^	−2.01 × 10^−3^−5.38 × 10^−3^

* Median and interquartile range are provided for runs of homozygosity, fraction of runs of homozygosity, and correlation between uniting gametes (F3). GWAS: Genome-wide association studies; IQR: interquartile range; ROH: runs of homozygosity; FROH: fraction of runs of homozygosity; CLL: chronic lymphocytic leukemia; DLBCL: diffuse large B-cell lymphoma; FL: follicular lymphoma; MZL: marginal zone lymphoma.

**Table 2.** Risk of NHL subtypes associated with measures of genome-wide homozygosity, FROH and F3*

	FROH					F3				
Subtype	β	SE	*P*-value	*I* ^2^	*P*_het_	β	SE	*P*-value	*I* ^2^	*P*_het_
CLL	21.14	4.41	1.59 × 10^−6^	0.0%	0.42	27.46	6.51	2.44 × 10^−5^	49.7%	0.11
DLBCL	0.04	10.89	1.0	72.5%	0.01	1.96	9.57	0.84	82.4%	0.001
FL	11.39	5.82	0.02	5.3%	0.37	13.19	8.01	0.10	64.2%	0.04
MZL	−0.87	7.88	0.91			6.40	5.20	0.22		

* Estimates of the log odds (β), standard error (SE), and *P*-value are provided for the association between FROH and F3 and each subtype, adjusted for age, sex (except UCSF1/NHS), percentage of missing SNPs, and principal components and combined using random effects meta-analysis. The *I*^2^ statistic provides an estimate of heterogeneity in association estimates across GWAS, and *P*_het_ is the *P*-value for heterogeneity among studies. FROH: Fraction of runs of homozygosity; NHL: non-Hodgkin lymphoma; CLL: chronic lymphocytic leukemia; DLBCL: diffuse large B-cell lymphoma; FL: follicular lymphoma; MZL: marginal zone lymphoma.
