# Supplementary material for: Genome-wide homozygosity and risk of four non-Hodgkin lymphoma subtypes
Source: J Transl Genet Genom. Author manuscript; Available in PMC 2021 Oct 6. (PMC8494431; doi:10.20517/jtgg.2021.08)
Supplement: Supplementary Tables [file NIHMS1720209-supplement-Supplementary_Tables.docx]

**Supplementary Tables**

| **Supplementary Table 1. Sample size and genotype arrays used for each NHL GWAS** | | | | | | | |
| --- | --- | --- | --- | --- | --- | --- | --- |
|  | **No. of Subjects in Analysis** | | | | |  |  |
| **Study** | **CLL** | **DLBCL** | **FL** | **MZL** | **Controls** | **Genotyping Platform** | **No. of Autosomal SNPs** |
| NCI^*^ | 2,140 | 2,621 | 2,085 | 808 | 6,105 | OmniExpress/Omni2.5 | 554,315 |
| GELA/EPIC |  | 547 |  |  | 525 | HumanHap 610K/660W | 486,277 |
| GEC | 387 |  |  |  | 294 | Affymetrix 6.0 | 604,007 |
| Mayo |  | 393 |  |  | 172 | HumanHap 660W | 492,020 |
| SCALE |  |  | 371 |  | 790 | HumanHap 317K | 289,632 |
| UCSF2^*^ | 213 | 253 | 209 |  | 745 | HumanCNV370-Duo | 289,900 |
| UCSF1/NHS |  |  | 119 |  | 340 | OmniExpress | 554,430 |
| Utah | 321 |  |  |  | 403 | HumanHap 610K | 483,331 |
| Total | 3,061 | 3,814 | 2,784 | 808 | 9,374 |  |  |
| ^*^The same set of controls was used for the different NHL subtypes in the study; however, quality control metrics were applied separately to each set of cases and controls, resulting in small differences in the number of controls and SNPs in the analysis. | | | | | | | |

| **Supplementary Table 2. Characteristics of cases and controls included in each NHL subtype analysis**   \|  \| **No. of Subjects** \| \| **% Female** \| \| **Mean Age (± SD)** \| \| \| --- \| --- \| --- \| --- \| --- \| --- \| --- \| \| **Study** \| **Cases** \| **Controls** \| **Cases** \| **Controls** \| **Cases** \| **Controls** \| \| **CLL** \|  \|  \|  \|  \|  \|  \| \| NCI NHL \| 2,140 \| 6,105 \| 44.4% \| 27.3% \| 65.6 (±9.7) \| 66.0 (±10.4) \| \| USCF2 \| 213 \| 746 \| 36.2% \| 42.4% \| 63.9 (±11.2) \| 61.5 (±13.0) \| \| GEC \| 387 \| 294 \| 34.9% \| 36.7% \| 61.3 (±11.1) \| 62.5 (±11.2) \| \| Utah \| 321 \| 403 \| 40.2% \| 43.9% \| 63.0 (±10.3) \| 64.1 (±10.8) \| \| **FL** \|  \|  \|  \|  \|  \|  \| \| NCI NHL \| 2,085 \| 6,105 \| 54.2% \| 27.3% \| 60.3 (±12.1) \| 66.0 (±10.4) \| \| USCF2 \| 209 \| 745 \| 47.4% \| 42.4% \| 60.9 (±11.9) \| 61.5 (±13.0) \| \| SCALE \| 371 \| 790 \| 50.4% \| 68.2% \| 57.0 (±9.5) \| 50.8 (±11.6) \| \| UCSF1/NHS \| 119 \| 340 \| 48.7% \| 100.0% \| 58.8 (±12.9) \| 62.6 (±8.6) \| \| **DLBCL** \|  \|  \|  \|  \|  \|  \| \| NCI NHL \| 2,621 \| 6,105 \| 51.1% \| 27.3% \| 60.7 (±14.0) \| 66.0 (±10.4) \| \| USCF2 \| 253 \| 745 \| 44.3% \| 42.4% \| 62.4 (±13.9) \| 61.5 (±13.0) \| \| Mayo \| 393 \| 172 \| 47.3% \| 61.6% \| 61.7 (±14.4) \| 51.1 (±13.8) \| \| GELA/EPIC \| 547 \| 525 \| 43.0% \| 55.1% \| 58.7 (±16.7) \| 58.5 (±9.5) \| \| **MZL** \|  \|  \|  \|  \|  \|  \| \| NCI NHL \| 808 \| 6,102 \| 59.7% \| 27.3% \| 64.0 (±12.1) \| 66.0 (±10.4) \| |
| --- | --- | --- | --- | --- | --- | --- | --- | --- | --- | --- | --- | --- | --- | --- | --- | --- | --- | --- | --- | --- | --- | --- | --- | --- | --- | --- | --- | --- | --- | --- | --- | --- | --- | --- | --- | --- | --- | --- | --- | --- | --- | --- | --- | --- | --- | --- | --- | --- | --- | --- | --- | --- | --- | --- | --- | --- | --- | --- | --- | --- | --- | --- | --- | --- | --- | --- | --- | --- | --- | --- | --- | --- | --- | --- | --- | --- | --- | --- | --- | --- | --- | --- | --- | --- | --- | --- | --- | --- | --- | --- | --- | --- | --- | --- | --- | --- | --- | --- | --- | --- | --- | --- | --- | --- | --- | --- | --- | --- | --- | --- | --- | --- | --- | --- | --- | --- | --- | --- | --- | --- | --- | --- | --- | --- | --- | --- | --- | --- | --- | --- | --- | --- | --- |

| **Supplementary Table 3. Individual GWAS and combined association results for the risk of each NHL subtype with FROH and F3** | | | | | | | | | | | |
| --- | --- | --- | --- | --- | --- | --- | --- | --- | --- | --- | --- |
|  |  | **FROH** | | | | | **F3** | | | | |
|  | **Study** | **β** | **SE** | **p-value** | **I^2^** | **p_het_** | **β** | **SE** | **p-value** | **I^2^** | **p_het_** |
| **CLL** | NCI | 18.85 | 4.73 | 6.73x10^-5^ |  |  | 27.45 | 3.47 | 2.82x10^-15^ |  |  |
|  | GEC | 50.61 | 19.64 | 0.01 |  |  | 33.84 | 10.61 | 0.001 |  |  |
|  | UCSF2 | 20.81 | 21.81 | 0.34 |  |  | 43.03 | 12.16 | 4.01x10^-4^ |  |  |
|  | Utah | 33.78 | 21.83 | 0.12 |  |  | -0.41 | 13.99 | 1.0 |  |  |
|  | *Combined* | 21.1 | 4.41 | 1.59x10^-6^ | 0.0% | 0.42 | 27.5 | 6.51 | 2.44x10^-5^ | 49.7% | 0.11 |
| **DLBCL** | NCI | 14.17 | 4.78 | 0.003 |  |  | 14.12 | 3.35 | 2.55x10^-5^ |  |  |
|  | Mayo | 7.96 | 35.21 | 0.82 |  |  | -6.15 | 19.04 | 0.75 |  |  |
|  | UCSF2 | -3.98 | 20.47 | 0.85 |  |  | 12.41 | 11.83 | 0.29 |  |  |
|  | GELA/EPIC | -15.71 | 7.80 | 0.04 |  |  | -15.53 | 6.53 | 0.02 |  |  |
|  | *Combined* | 0.0 | 10.89 | 1.0 | 72.5% | 0.01 | 2.0 | 9.57 | 0.84 | 82.4% | 0.001 |
| **FL** | NCI | 6.72 | 5.12 | 0.19 |  |  | 5.42 | 3.62 | 0.13 |  |  |
|  | UCSF1/NHS | 5.15 | 34.26 | 0.88 |  |  | -18.04 | 22.07 | 0.41 |  |  |
|  | UCSF2 | 35.13 | 18.54 | 0.06 |  |  | 21.75 | 11.68 | 0.06 |  |  |
|  | SCALE | 20.05 | 10.45 | 0.06 |  |  | 28.64 | 9.04 | 0.002 |  |  |
|  | *Combined* | 11.4 | 5.82 | 0.02 | 5.3% | 0.37 | 13.2 | 8.01 | 0.10 | 64.2% | 0.04 |
| **MZL** | NCI | -0.87 | 7.88 | 0.91 |  |  | 6.4 | 5.2 | 0.22 |  |  |
| ^*^Estimates of the log odds (β), standard error (SE), and p-value are provided for the association between FROH and F3 and each subtype, adjusted for age, sex (except UCSF1/NHS), percentage of missing SNPs, and principal components, and combined using random effects meta-analysis. The I² statistic provides an estimate of heterogeneity in association estimates across GWAS, and Phet is p-value for heterogeneity among studies. | | | | | | | | | | | |

| **Supplementary Table 4. Sensitivity analysis of the risk of each NHL subtype associated with FROH and F3 after removing individual studies from the meta-analysis^*^** | | | | | | | | |
| --- | --- | --- | --- | --- | --- | --- | --- | --- |
|  |  | **FROH** | | |  | **F3** | | |
|  | Study Removed^†^ | **β** | **SE** | p-value |  | **β** | **SE** | p-value |
| CLL | NCI | 36.19 | 12.13 | 0.003 |  | 26.58 | 12.14 | 0.03 |
|  | GEC | 19.57 | 4.52 | 1.49x10^-5^ |  | 25.08 | 9.32 | 0.007 |
|  | UCSF2 | 26.29 | 8.98 | 0.003 |  | 23.83 | 7.42 | 0.001 |
|  | Utah | 23.25 | 7.62 | 0.002 |  | 29.09 | 3.19 | 6.25x10^-20^ |
|  |  |  |  |  |  |  |  |  |
| DLBCL | NCI | -13.31 | 7.13 | 0.06 |  | -4.68 | 9.75 | 0.63 |
|  | Mayo | -0.78 | 12.08 | 0.95 |  | 3.36 | 10.89 | 0.76 |
|  | UCSF2 | 0.83 | 13.11 | 0.95 |  | -1.37 | 12.30 | 0.91 |
|  | GELA/EPIC | 13.14 | 4.62 | 0.004 |  | 13.43 | 3.18 | 2.34x10^-5^ |
|  |  |  |  |  |  |  |  |  |
| FL | NCI | 22.46 | 8.80 | 0.01 |  | 18.22 | 10.37 | 0.08 |
|  | UCSF1/NHS | 14.14 | 7.04 | 0.04 |  | 16.67 | 8.41 | 0.05 |
|  | UCSF2 | 9.23 | 4.56 | 0.04 |  | 10.35 | 10.30 | 0.32 |
|  | SCALE | 10.12 | 6.72 | 0.13 |  | 7.38 | 6.97 | 0.29 |
|  |  |  |  |  |  |  |  |  |
| ^*^Estimates of the log odds (β), standard error (SE), and p-value are provided for the association between FROH and F3 and each subtype, adjusted for age, sex (except UCSF1/NHS), percentage of missing SNPs, and principal components, and combined using random effects meta-analysis.  ^†^This column indicates the specific study removed from the meta-analysis. The estimates presented are from the random effects meta-analysis after excluding this study. | | | | | | | | |
